# Synergistic Fe–Ni dual-atom sites on hollow carbon enabling high-performance rechargeable zinc–air batteries

**DOI:** 10.1039/d5sc07448g

**Published:** 2025-11-25

**Authors:** Yue Wang, Jianhua Wang, Xueting Feng, Guanzhen Chen, Xusheng Wang, Tao Gan, Xing Fan, Haiping Lin, Yunhu Han

**Affiliations:** a State Key Laboratory of Flexible Electronics (LoFE), Institute of Advanced Materials (IAM), School of Chemistry and Life Sciences, Nanjing University of Posts & Telecommunications Nanjing China iamyhhan@njupt.edu.cn guanzhenchen123@163.com; b Frontiers Science Center for Flexible Electronics, Institute of Flexible Electronics (IFE), Northwestern Polytechnical University Xi'an China; c School of Physics and Information Technology, Shaanxi Normal University Xi'an China; d Shanghai Synchrotron Radiation Facilities, Shanghai Institute of Applied Physics, Chinese Academy of Sciences Shanghai China gant@sari.ac.cn; e State Key Laboratory of Bio-based Fiber Materials, School of Materials Science and Engineering, Zhejiang Sci-Tech University Hangzhou China; f Center for Carbon-based Electronics and Key Laboratory for the Physics and Chemistry of Nanodevices, School of Electronics, Peking University Beijing China xingf@pku.edu.cn

## Abstract

The advancement of rechargeable zinc–air batteries (ZABs) hinges critically on the development of efficient and durable bifunctional oxygen electrocatalysts. Herein, we report an atomically dispersed Fe–Ni bifunctional catalyst loaded on a hollow carbon framework (FeNi-hCN) through a density difference-assisted strategy. This unique architecture, leveraging the synergistic interplay between Fe and Ni atoms and the advantageous properties of the hollow carbon support, endows the catalyst with exceptional bifunctional oxygen electrocatalytic activity: a half-wave potential (*E*_1/2_) of 0.91 V for the oxygen reduction reaction (ORR) and overpotential of only 330 mV at 10 mA cm^−2^ for the oxygen evolution reaction (OER). Remarkably, the catalyst demonstrates outstanding stability, retaining its activity after 100 000 accelerated degradation test (ADT) cycles and 240 hours of continuous OER operation. When deployed as the air cathode in aqueous ZABs, this catalyst achieves a high peak power density of 212 mW cm^−2^ and stable cycling for 560 hours, outperforming a Pt/C + RuO_2_ combination. Density functional theory (DFT) calculations elucidate that the Fe–Ni dual sites synergistically lower the adsorption energy of the critical *OOH intermediate, thereby reducing the overall energy barriers for both ORR and OER pathways. This density difference-assisted method also works for other MOFs like UiO-66 and HKUST-1, enabling diverse high-performance carbon-supported catalysts.

## Abstract 2

A Fe–Ni dual-atom catalyst on a density-difference carbon support enables durable oxygen electrocatalysis (100 000 ORR cycles; 240 h OER). The resulting Zn–air battery achieves a 212 mW cm^−2^ peak power density and 560-hour cycling stability.

## Graphical abstract

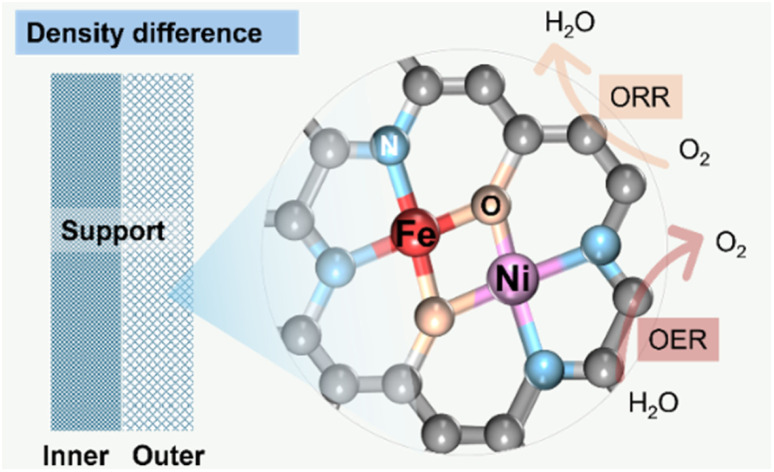

## Introduction

Zinc–air batteries (ZABs) represent promising candidates for next-generation sustainable energy storage due to their high theoretical energy density, environmental benignity, and abundant resource availability.^1–3^ Nevertheless, their practical implementation is constrained by the sluggish reaction kinetics of both oxygen reduction (ORR) and oxygen evolution (OER) at the air cathode.^4,5^ While noble metal-based catalysts exhibit high activity for individual reactions, their scalable deployment is hindered by three intrinsic limitations: insufficient bifunctional activity for ORR/OER cycling, high cost, and limited long-term stability.^6,7^ Furthermore, conventional hybrid catalysts with spatially separated active sites exacerbate interfacial resistance and accelerate structural degradation due to incompatible reaction environments.^8^ Consequently, developing cost-effective non-precious metal catalysts with integrated bifunctionality redox oxygen electrocatalysis and enhanced durability constitutes a critical pathway toward overcoming ZABs' technological barriers.

Atomic-level dispersed transition metal–nitrogen–carbon (M–N–C) materials have emerged as a frontier in research on bifunctional electrocatalysts due to their tunable electronic structure, theoretically maximum atomic utilization efficiency, and well-defined active sites.^9,10^ However, although single-atom catalysts (such as Fe–N_4_ or Co–N_4_) exhibit excellent performance in either the oxygen reduction reaction (ORR) or oxygen evolution reaction (OER) individually, their bifunctional activity remains constrained by three inherent limitations: single active sites struggle to optimize ORR/OER reaction pathways synergistically, adsorption strengths for OOH/OH intermediates cannot be balanced simultaneously, and fluctuations in metal valence states during charge–discharge cycles lead to deactivation.^11,12^ To overcome this bottleneck, constructing heteronuclear bimetallic sites (such as Fe/Co–N_4_) and then bidirectionally regulating intermediate adsorption energy through electronic coupling effects could theoretically enable the development of catalysts with both bifunctional activity and stability.^12–15^ In addition, structural defects in the support further constrain the final performance. The dense pore structure of traditional carbon substrates results in low active site exposure rates, and slow oxygen diffusion rates cause mass transfer polarization, leading to a sharp decline in performance under actual working conditions.^16–18^ Therefore, the development of non-precious metal atomic-level catalysts with the following characteristics is the key to breaking the deadlock: stable anchoring of heteronuclear bimetallic active centers, synergistic optimization of ORR/OER adsorption energy, and hierarchical porous supports to promote mass transfer and active site exposure.

To address the above challenges, a structural density difference-assisted strategy was proposed in this study to successfully construct nitrogen-doped hollow carbon-based catalysts (FeNi-hCN) loaded with Fe–Ni bi-atomic sites. Synchrotron X-ray absorption spectroscopy (XAS) and spherical aberration corrected electron microscopy (HAADF-STEM) reveal that Fe and Ni atoms are embedded in the carbon skeleton through bridged N/O-atom configurations, and the atomic dispersion of the diatomic sites as well as the strong electronic coupling effect are also confirmed. In addition, the hollow hierarchical porous structure achieved by using structural density difference induction not only enhances the density of active sites but also promotes reactant transport and gas diffusion. Electrochemical evaluation reveals superior bifunctionality: ORR half-wave potential (*E*_1/2_) = 0.91 V *vs.* RHE, exceeding Pt/C by 38 mV; OER overpotential (*η*_10_) = 330 mV at 10 mA cm^−2^. Moreover, the decay of the activity after 100 000 ORR cycles and 240 h of OER operation was negligible. The liquid ZABs assembled based on this catalyst reached a peak power density of 211.62 mW cm^−2^, with a voltage efficiency decay rate as low as 0.05% h^−1^ after 560 h of charge/discharge cycling. Density functional theory (DFT) calculations further indicate the mechanism of synergy between Fe–Ni bimetallic sites, which significantly reduces the ORR/OER reaction energy barrier. This study provides a new paradigm for the design of atomic-scale bimetallic catalysts and their application in high-efficiency energy devices.

## Results and discussion

**Fig. 1:**
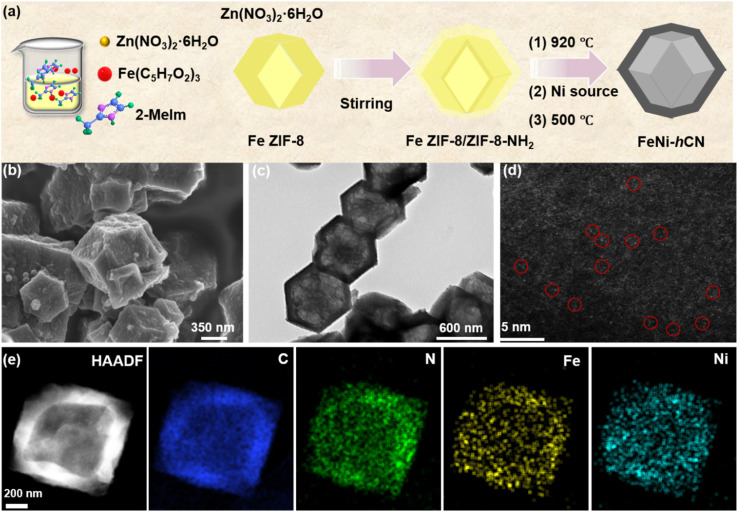
(a) Schematic illustration of the fabrication process of the FeNi-hCN catalyst, (b) SEM image, (c) TEM image, and (d) AC HAADF-STEM image of the FeNi-hCN, (e) HAADF-STEM images of the FeNi-hCN and the corresponding EDS elemental mapping.

The FeNi-hCN electrocatalyst was synthesized through a multi-step process (Fig. 1a). Initially, Fe/ZIF-8 cores were prepared *via* room-temperature stirring, followed by epitaxial growth of ZIF-8-NH_2_ to form Fe/ZIF-8@ZIF-8-NH_2_ core–shell precursors. Subsequent pyrolysis under Ar at 900 °C induced Zn volatilization generating mesoporous defects, while partial Fe migration from the core to outer-shell vacancies occurred, yielding Fe-hCN intermediates. This structural evolution is primarily driven by the density difference between the core and shell layers, which guides the preferential volatilization of zinc during pyrolysis and the subsequent migration of iron. Ni was then introduced through thermal impregnation with subsequent low-temperature pyrolysis (350 °C) enabling atomic anchoring, followed by acid leaching to obtain the final Fe–Ni dual-atom catalyst. Structural characterization confirms that FeNi-hCN preserved rhombic dodecahedral morphology (Fig. 1b, c and S1) with distinct bilayer contrast at edges. This structural morphology originates from the Fe/ZIF-8@ZIF-8-NH_2_ precursor and is preserved during pyrolysis and acid treatment, owing to the structural rigidity of the derived nitrogen-doped carbon framework. Atomic-resolution HAADF-STEM (Fig. 1d) revealed isolated diatomic pairs (Fe–Ni distance: 2.3–2.8 Å) indicated by red circles.^19–22^ Elemental mapping (Fig. 1e) demonstrated core–shell spatial distribution of C, N, Fe, and Ni. Control samples (solid FeNi-CN from Fe/ZIF-8, Fig. S2 and S3) lack this bilayer architecture. Powder X-ray diffraction (PXRD) analysis of the FeNi-hCN and hCN catalysts revealed a characteristic broad peak centered at 44.1°, corresponding to the (101) plane of carbon (Fig. S4).^18^ No distinct diffraction peaks attributable to crystalline Fe or Ni phases were observed. This absence of metal phase signatures in PXRD is consistent with the findings from AC HAADF-STEM. To accurately quantify the elemental composition of the FeNi-hCN catalyst, inductively coupled plasma mass spectrometry (ICP-MS) was employed. The measured Fe and Ni contents were determined to be 1.7 wt% and 1.3 wt%, respectively (Table S1), yielding an Fe/Ni atomic ratio of nearly 1 : 1.

**Fig. 2:**
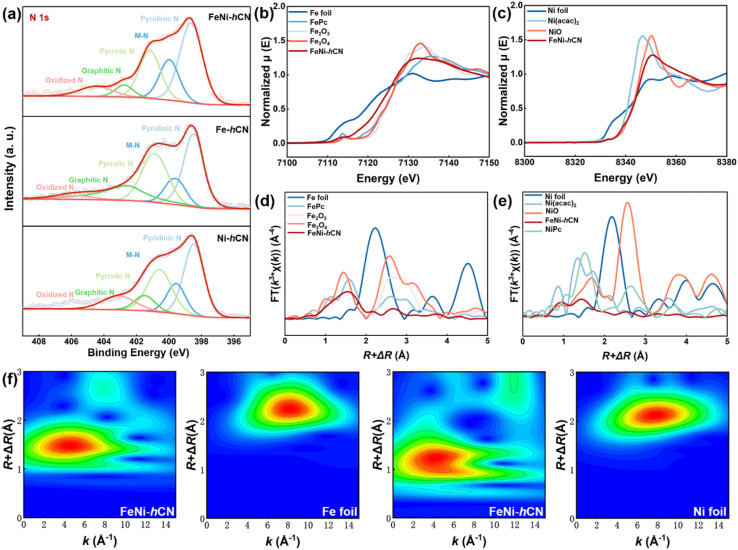
(a) High-resolution XPS spectra of N 1s for the FeNi-hCN, Fe-hCN and Ni-hCN catalysts. (b) Fe K-edge XANES spectra. (c) Ni K-edge XANES spectra. (d) Fe K-edge *k*^3^-weighted Fourier Transform (FT) EXAFS spectra. (e) Ni K-edge *k*^3^-FT-EXAFS spectra. (f) WT EXAFS contour plots of Fe K-edge for FeNi-hCN and Fe foil, Ni K-edge for FeNi-hCN, and Ni foil.

X-ray photoelectron spectroscopy (XPS) analysis was performed on FeNi-hCN to characterize its surface composition and chemical states. As shown in Fig. 2a, the high-resolution N 1s spectrum of the catalyst was deconvoluted into five distinct components corresponding to pyridinic N (398.6 eV), metal-coordinated N (M–N, 399.9 eV), pyrrolic N (401.6 eV), graphitic N (402.7 eV), and oxidized N (404.4 eV).^23–25^ The presence of the M–N component confirms the direct anchoring of Fe/Ni atoms to the nitrogen-doped carbon support *via* chemical bonding.^26–28^ Quantitative analysis of the peak areas (Table S2) revealed that the FeNi-hCN bimetallic catalyst exhibits a higher ratio of pyridinic N to M–N species compared to its monometallic counterparts (Fe-CN and Ni-CN). This observation suggests that the coexistence of Fe and Ni sites influences the local nitrogen coordination environment within the carbon nitride matrix.^16,23,29^ The coexistence of Fe and Ni in FeNi-hCN was further confirmed by Fe 2p and Ni 2p spectra (Fig. S5 and S6). Notably, the binding energies of the primary Fe 2p peaks in FeNi-hCN exhibited a positive shift relative to Fe-CN, while the primary Ni 2p peaks showed a negative shift relative to Ni-CN. These binding energy shifts indicate a strong electronic coupling between Fe and Ni atoms in the bimetallic system, consistent with partial electron transfer from Fe to Ni.^30–32^

The coordination environment and electronic structure of FeNi-hCN at the atomic level were further elucidated by X-ray absorption fine structure (XAFS) measurements. Fig. 2b shows the Fe K-edge X-ray absorption near-edge structure (XANES) spectra. The absorption edge energy of FeNi-hCN locates between Fe foil and iron phthalocyanine (FePc), indicating an average Fe valence state between 0 and +2. Similarly, the Ni K-edge XANES spectrum (Fig. 2c) reveals an average Ni valence state close to +2. The observed valence states are consistent with the XPS findings. Fourier transform (FT) *k*^3^-weighted extended X-ray absorption fine structure (EXAFS) spectra for the Fe K-edge in FeNi-hCN are presented in Fig. 2d. The dominant peak at ∼1.53 Å is assigned to first-shell coordination of Fe to light atoms (N/O).^20,33,34^ Meanwhile, a smaller peak is only observed at ∼2.13 Å, which is similar to that of the isolated single Fe atoms and unlike the Fe–Fe coordination form in Fe foil and attributed to Fe–Ni non-interacting coordination.^19,32^ Similarly, the FT-EXAFS spectrum at the Ni K-edge (Fig. 2e) exhibits a primary peak at ∼1.44 Å, assigned to Ni–N/O first-shell coordination, and a secondary peak at ∼2.06 Å attributed to Ni–Fe coordination. This signal is compatible with the bimetallic pair configuration observed by AC HAADF-STEM, providing structural evidence supporting a bimetallic site. EXAFS fitting results (Fig. S7 and S8; Table S3) further support this analysis, yielding average coordination numbers for Fe–N/O (2.90), Fe–Ni (1.26), Ni–N/O (3.71), and Ni–Fe (0.15). Wavelet transform (WT) EXAFS analysis (Fig. 2f and S9) was performed to resolve atomic arrangements in *R*-space. The contour plots for Fe and Ni K-edges in FeNi-hCN exhibit maximum intensities at approximately 4.0 Å^−1^ and 4.2 Å^−1^ in *k*-space, respectively. Critically, the absence of intensity maxima characteristic of Fe-foil and Ni-foil references confirms the atomic dispersion of both Fe and Ni species.^35,36^ Collectively, these XAFS results provide strong evidence for the existence of a bimetallic single-atom structure in FeNi-hCN.

**Fig. 3:**
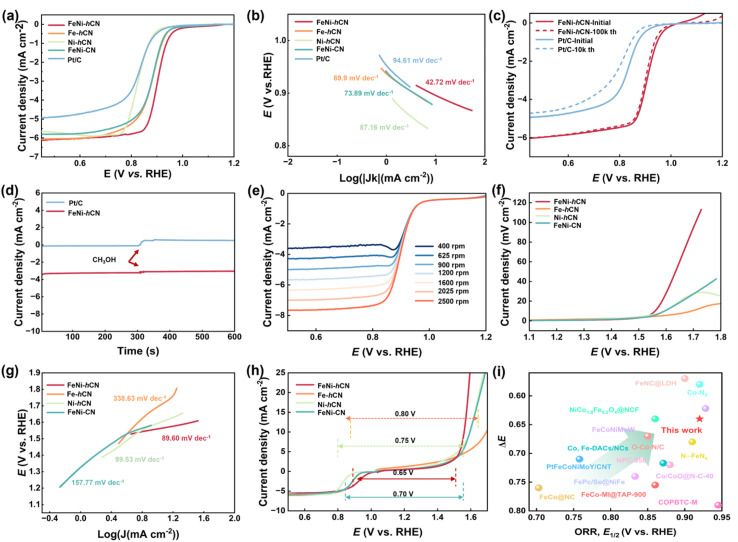
(a) LSV curves of samples measured in O_2_-saturated 0.1 M KOH, (b) Tafel plots, (c) comparison of the potential cycling performance of the FeNi-hCN and commercial Pt/C in 0.1 M KOH electrolyte. (d) Methanol tolerance of the FeNi-hCN and commercial Pt/C catalysts in O_2_ saturated 0.1 M KOH media. (e) LSV curves at different rotating rates. (f) LSV polarization curves of the OER, (g) Tafel curves for FeNi-hCN and the control catalysts, (h) the overall LSV polarization curves and Δ*E* of the FeNi-hCN, Fe-hCN, Ni-hCN and FeNi-CN catalysts. (i) Comparison of samples in this work with those of recently reported catalysts.

The oxygen reduction reaction (ORR) electrocatalytic performance of FeNi-hCN was evaluated in oxygen-saturated 0.1 M KOH using a standard three-electrode configuration. Linear sweep voltammetry (LSV) curves (Fig. 3a) reveal that FeNi-hCN exhibits a more positive half-wave potential (*E*_1/2_ = 0.91 V *vs.* RHE) compared to the commercial Pt/C benchmark (*E*_1/2_ = 0.83 V *vs.* RHE) and control catalysts. While the monometallic Fe-hCN (*E*_1/2_ = 0.88 V *vs.* RHE) and FeNi-CN (*E*_1/2_ = 0.879 V *vs.* RHE) display similar alkaline ORR activity to each other, Ni-hCN performance is significantly poorer (*E*_1/2_ = 0.83 V *vs.* RHE). Kinetic current densities (*j*_k_) derived from mass-transport correction further highlight the superior activity of FeNi-hCN (Fig. S10). At 0.9 V *vs.* RHE, the *j*_k_ for FeNi-hCN reaches 10.28 mA cm^−2^, significantly exceeding those of Fe-hCN (4.35 mA cm^−2^) and Ni-hCN (1.10 mA cm^−2^). Similarly, at 0.85 V *vs.* RHE, FeNi-hCN achieves a *j*_k_ of 25.0 mA cm^−2^, markedly higher than the control catalysts. The enhanced kinetic current density demonstrates the favorable ORR kinetics of FeNi-hCN. Tafel slopes (Fig. 3b) show that FeNi-hCN possesses a lower Tafel slope (42.72 mV dec^−1^) than commercial Pt/C (94.61 mV dec^−1^), indicating improved reaction kinetics. Catalyst stability, a critical parameter for practical application, was assessed *via* accelerated durability testing (ADT) involving 100 000 potential cycles (Fig. 3c).^37,38^ FeNi-hCN exhibited minimal degradation in ORR activity after this rigorous testing. Post-ADT aberration-corrected high-angle annular dark-field scanning transmission electron microscopy (AC HAADF-STEM) images (Fig. S11) confirmed no observable aggregation of Fe or Ni species, corroborating the structural stability. Rotating ring-disk electrode (RRDE) measurements (Fig. S12) indicate that the ORR on FeNi-hCN proceeds predominantly *via* a four-electron pathway, with low hydrogen peroxide yield, demonstrating high selectivity.^20,23,39,40^ Furthermore, FeNi-hCN exhibited excellent methanol tolerance, maintaining its ORR activity in the presence of methanol (Fig. 3d). LSV measurements at varied rotation rates (Fig. 3e) show that the limiting current density of FeNi-hCN increases with rotation speed. This dependence on oxygen diffusion rate confirms that the reaction kinetics are not solely limited by electron transfer but also by mass transport under these conditions.^41–43^

The oxygen evolution reaction (OER) activity of the catalyst is also crucial for practical Zn–air batteries (ZABs) and water splitting applications. Linear sweep voltammetry (LSV) curves measured in N_2_-saturated 1 M KOH (Fig. 3f and S13) demonstrate that FeNi-hCN achieves a current density of 10 mA cm^−2^ at an overpotential (*η*) of 330 mV, indicating superior OER activity compared to control catalysts. Furthermore, FeNi-hCN exhibits excellent stability in alkaline media, showing negligible activity decay after 240 hours of chronopotentiometric testing at a constant current density (Fig. S14). Tafel analysis provides insights into the OER kinetics (Fig. 3g). The Tafel slopes follow the order: FeNi-hCN (89.6 mV dec^−1^) < Ni-hCN (99.53 mV dec^−1^) < FeNi-CN (157.77 mV dec^−1^) < Fe-hCN (338.63 mV dec^−1^). The lower Tafel slope of FeNi-hCN signifies more favorable OER kinetics.

The bifunctional activity, essential for rechargeable ZABs, was quantified by the potential difference Δ*E* = *E*_*j*=10_(OER) − *E*_1/2_(ORR), where *E*_*j*=10_ is the potential at 10 mA cm^−2^ OER current density and *E*_1/2_ is the ORR half-wave potential. A smaller Δ*E* indicates reduced potential hysteresis between the ORR and OER, signifying better bifunctional catalyst performance. As shown in Fig. 3h, FeNi-hCN exhibits the smallest Δ*E* value of 0.65 V, outperforming Fe-hCN (Δ*E* = 0.80 V), Ni-hCN (Δ*E* = 0.75 V), and FeNi-CN (Δ*E* = 0.70 V). This outstanding bifunctional performance of FeNi-hCN is competitive with, or superior to, many reported non-precious metal catalysts, as benchmarked in Fig. 3i and Table S4, highlighting its significant potential as a bifunctional oxygen electrocatalyst.

**Fig. 4:**
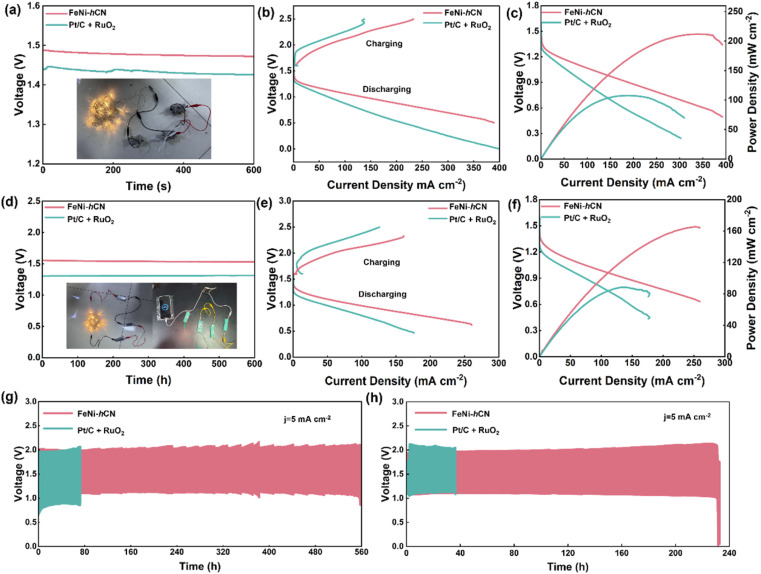
(a) Open-circuit voltage test results of liquid ZABs and three ZABs in series lighting up different colors of LEDs, (b) charge–discharge polarization curves, (c) discharge polarization and corresponding power density curves, (d) open-circuit voltage test results of flexible quasi-solid-state rechargeable ZABs, (e) charge–discharge polarization curves, (f) discharge polarization and corresponding power density curves, (g) cycling stability test of liquid alkaline ZABs with an air cathode of FeNi-hCN catalyst, (h) cycling stability test of flexible quasi-solid-state alkaline ZABs with an air cathode of FeNi-hCN catalyst.

Motivated by the promising bifunctional oxygen electrocatalytic activity of FeNi-hCN, its application as an air cathode catalyst in rechargeable Zn–air batteries (ZABs) was investigated. A liquid ZAB was assembled using catalyst-coated carbon paper (FeNi-hCN) as the air cathode, Zn foil as the anode, and an aqueous electrolyte comprising 6 M KOH and 0.2 M Zn(CH_3_COO)_2_. The FeNi-hCN-based ZAB exhibited a high open-circuit voltage (OCV) of 1.48 V (Fig. 4a and S15). Discharge–charge polarization curves (Fig. 4b) revealed that the FeNi-hCN-based cell possesses a smaller voltage gap between charge and discharge processes compared to the benchmark Pt/C + RuO_2_-based cell, indicating superior energy efficiency and rechargeability. Performance metrics further confirmed the advantage of FeNi-hCN: it delivered a peak power density of 211.62 mW cm^−2^ (Fig. 4c) and a specific capacity of 801.68 mAh g^−1^ (based on Zn consumption, Fig. S16), both exceeding the performance of the Pt/C + RuO_2_-based ZAB and many reported non-precious metal-based liquid ZABs (Fig. S17). Furthermore, the FeNi-hCN-based liquid ZAB demonstrated excellent cycling stability. It sustained over 560 hours of continuous charge–discharge cycling at a constant current density of 5 mA cm^−2^ (Fig. 4g), with minimal degradation in performance, confirming its practical potential for long-term operation.

The battery structure comprised FeNi-hCN-coated carbon cloth as the cathode, Zn foil as the anode, and a polyvinyl alcohol (PVA)–KOH gel electrolyte. The assembled flexible quasi-solid-state ZAB exhibited a high open-circuit voltage (OCV) of 1.55 V (Fig. 4d and S18). Demonstrating practical potential, three series-connected FeNi-hCN-based ZABs successfully powered a string of commercial light-emitting diodes (LEDs), and four batteries in series were capable of charging a mobile phone (Fig. 4d, inset). Discharge–charge polarization curves (Fig. 4e) revealed that the FeNi-hCN-based flexible ZAB possesses a significantly smaller voltage gap compared to its Pt/C + RuO_2_ counterpart, indicating superior energy efficiency for rechargeable operation. Performance evaluation (Fig. 4f) showed that the FeNi-hCN-based ZAB delivers a discharge plateau and achieves a peak power density of 165.32 mW cm^−2^. Furthermore, it provided a high specific capacity of 810.69 mAh g^−1^ (based on Zn, Fig. S19), confirming its substantial energy storage capability. The FeNi-hCN-based flexible quasi-solid-state ZAB also demonstrated excellent cycling stability. Over 220 hours of continuous charge–discharge cycling at a constant current density of 5 mA cm^−2^ (Fig. 4h), the battery exhibited minimal performance degradation. Compared with the reported non-precious metal-based flexible quasi-solid-state ZABs, the FeNi-hCN-based flexible ZAB showed an excellent performance (Fig. S20). Rate capability testing (Fig. S21) indicated robust performance across different current densities, with the voltage gap remaining comparable to the initial value even after operation at higher rates, highlighting its capability for high-power applications. Additionally, the battery-maintained functionality under mechanical stress, performing reliably after being folded to various angles (Fig. S22).

**Fig. 5:**
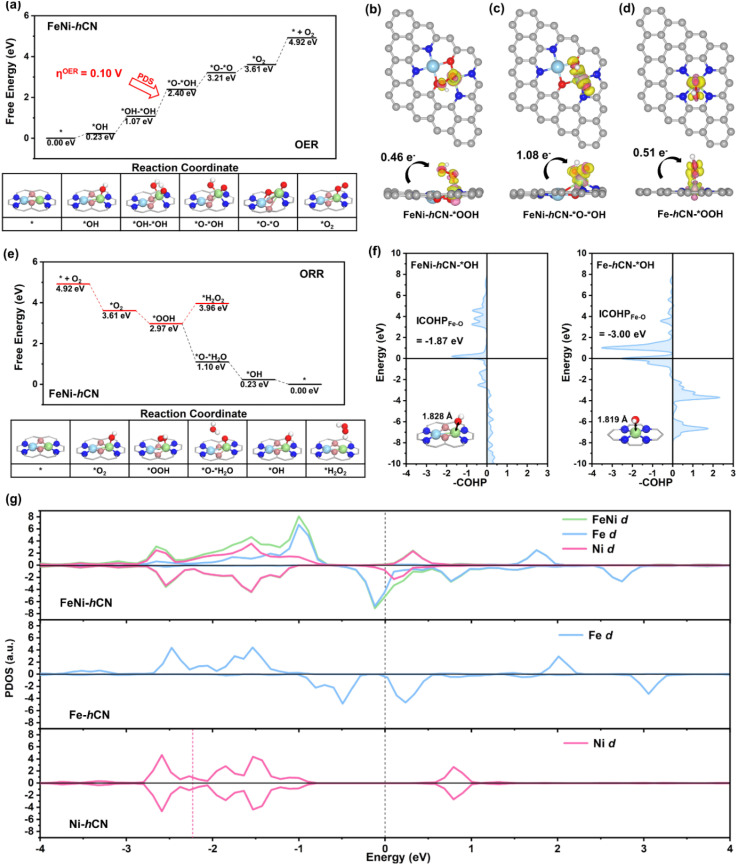
(a) Gibbs free energy diagram of the OPM-OER pathway on FeNi-hCN. Charge difference density isosurfaces (iso-surface value = 0.01 |*e*| Å^−3^) with electron accumulation (yellow) and depletion (pink) for: (b) *OOH adsorbed on FeNi-hCN, (c) *O–*OH adsorbed on FeNi-hCN, and (d) *OOH adsorbed on Fe-hCN. Charge transfer values (|*e*|) are annotated. (e) Gibbs free energy diagrams of ORR pathways on FeNi-hCN. (f) Crystal Orbital Hamilton Population (COHP) between analysis of the Fe–O bonds in FeNi-hCN-*OH and Fe-hCN-*OH, respectively. The Fermi level is set to 0.00 eV. (g) Projected density of states (PDOS) of metal d orbitals for FeNi-hCN, Fe-hCN and Ni-hCN. The Fermi level is set to 0.00 eV.

Density functional theory (DFT) calculations were employed to elucidate the synergistic mechanism of Fe–Ni bimetallic sites by comparing the electronic structures and behaviors of key oxygen-containing intermediates (*OH, *O, *OOH and *O–OH) on FeNi-hCN, Fe-hCN, and Ni-hCN catalysts (models based on Fig. S25). Gibbs free energy diagrams for the OER on all of these three catalysts are presented in Fig. 5a and S24. For the OER on FeNi-hCN, both the adsorbate evolution mechanism (AEM)^43^ (*OH → *O–*H_2_O → *OOH → *O_2_) and Oxide Path Mechanism (OPM)^44^ (*OH → *OH–*OH → *O–*OH → *O_2_) pathways were evaluated due to dual metal sites. For FeNi-hCN, the AEM exhibits a rate-determining step (RDS) (*O–*H_2_O → *OOH) with a high free energy uphill of 1.87 eV (OER overpotential *η* = 0.64 V; Fig. S24a). In contrast, *via* the OPM, the RDS (*OH–*OH → *O–*OH) shows a significantly reduced *η* of 0.10 V (Fig. 5a). Therefore, for FeNi-hCN, the OPM is thermodynamically favored over the AEM. This preference arises from stronger *O–*OH adsorption (charge transfer: 1.08 e^−^) compared to *OOH (0.46 e^−^), as confirmed by Bader charge analysis and differential charge density maps (Fig. 5b and c). Single-atom catalysts Fe-hCN (RDS: *O → *OOH, *η* = 0.70 V) and Ni-hCN (RDS: *OH → *O, *η* = 0.66 V) exhibit inferior AEM OER activity (Fig. S24b and c), demonstrating that the Fe–Ni synergy induces a mechanistic shift to the efficient OPM. Furthermore, this pathway is thought to be enabled by an inter-metal distance conducive to forming binuclear intermediates. The measured Fe–Ni distance of 2.57 Å in our work is consistent with this geometric principle, as it resides within the commonly cited range of ∼2.50–3.00 Å.^45–48^ Such a configuration appears highly favorable for coordinating key intermediates, most notably the *O–*OH species, which could explain the enhanced catalytic performance. For the ORR, Gibbs free energy diagrams for both the 2-electron (H_2_O_2_ formation) and 4-electron (H_2_O formation) pathways were calculated (Fig. 5e and S25). FeNi-hCN favors the 4-electron pathway (exothermic overall), with a lower RDS energy barrier for *OH reduction to H_2_O (0.23 eV) compared to Fe-hCN (0.03 eV). Crystal Orbital Hamilton Population (COHP) analysis^49^ further elucidated the impact of Fe–Ni synergy on *OH desorption kinetics (Fig. 5f). In comparison with Fe-hCN-*OH for Fe single atoms, FeNi-hCN exhibits a stronger antibonding state of Fe–O near the Fermi level. Meanwhile, the integrated COHP (ICOHP) value for *OH adsorption on FeNi-hCN (−1.87 eV) is significantly weaker than that on Fe-hCN (−3.00 eV), confirming stronger *OH binding on the latter. This fact aligns with the bond lengthening observed for FeNi-hCN-*OH, which exhibits a longer Fe–O bond length (1.828 Å) than Fe-hCN-*OH (1.819 Å). Furthermore, as shown in Fig. 5b and d, Bader charge analysis revealed fewer electrons transferred from the FeNi-hCN surface to adsorbed *OOH (0.46 e^−^) compared to the Fe-hCN surface (0.51 e^−^). These results provide robust evidence that the Fe–Ni dual-atom synergistic effect effectively modulates the adsorption strength of key oxygen-containing intermediates (*e.g.*, *OH, *OOH), thereby optimizing ORR activity. Ni-hCN demonstrates the poorest ORR activity due to difficult O_2_ adsorption (Fig. S23b). To elucidate the electronic origin of the superior OER/ORR bifunctionality of FeNi-hCN, projected density of states (PDOS) analysis of metal d-orbitals for FeNi-hCN, Fe-hCN, and Ni-hCN was performed (Fig. 5g). Notably, in contrast to Fe-hCN and Ni-hCN, which exhibit negligible d-orbital states near the Fermi level, FeNi-hCN possesses significant d-orbital states near the Fermi level. This feature originates from Fe 3d–Ni 3d orbital hybridization, which restructures the electronic landscape near the Fermi level and enhances charge transfer capability at bimetallic sites – facilitating both electron donation to adsorbed species and stabilization of electron-deficient intermediates. Consequently, this electronic configuration optimizes the adsorption energetics of key reaction intermediates, thereby enhancing the bifunctional catalytic activity of FeNi-hCN.

To assess the broader applicability of the hollow carbon nanostructure synthesis strategy, we applied the methodology to UiO-66 and HKUST-1 metal–organic frameworks (MOFs) as alternative precursors. Transmission electron microscopy (TEM) characterization (Fig. S26 and S27) reveals a distinct core–shell morphology in both systems. This observation is consistent with the successful formation of a secondary zeolitic imidazolate framework (ZIF) layer templated on the initial MOF particles.

## Conclusions

This work demonstrates that atomically dispersed Fe–Ni dual sites anchored on a hollow carbon framework (FeNi-hCN), synthesized *via* a density difference-assisted strategy, achieve exceptional bifunctional oxygen electrocatalysis through synergistic electronic coupling and enhanced mass transport. The catalyst exhibits outstanding activity with an ORR half-wave potential of 0.91 V *vs.* RHE and a low OER overpotential of 330 mV at 10 mA cm^−2^, alongside remarkable stability evidenced by 100 000 accelerated ORR cycles and 240-hour OER operation. When integrated into aqueous Zn–air batteries, FeNi-hCN delivers a peak power density of 212 mW cm^−2^ and sustains 560-hour continuous cycling, surpassing Pt/C + RuO_2_. Density functional theory calculations reveal that Fe–Ni cooperation optimizes *OOH intermediate adsorption, reducing energy barriers for both the ORR and OER. Crucially, the density-guided methodology proven universally shows potential applicability to MOFs (*e.g.*, UiO-66, HKUST-1), establishing a versatile platform for designing high-performance bimetallic electrocatalysts for advanced energy technologies.

## Author contributions

Y. Wang, J. H. Wang and X. T. Feng contributed equally to this work. Y. Wang designed and performed the research on material synthesis, data analysis and manuscript writing. Y. H. Han developed the idea for the study. J. H. Wang, X. T. Feng, G. Z. Chen, X. S. Wang, T. Gan, X. Fan, and H. P. Lin assisted with the electrochemical tests and data analyses. All the authors contributed to the writing and revisions of the manuscript.

## Conflicts of interest

There are no conflicts to declare.

## Supplementary Material

SC-017-D5SC07448G-s001

## Data Availability

The authors confirm that the data supporting the findings of this study are available within the article or its supplementary information (SI). Supplementary information: complete experimental procedures, supporting characterization techniques, data and the details for the prepared catalysts are provided. See DOI: https://doi.org/10.1039/d5sc07448g.
