# Supplementary material for: Synergistic Fe–Ni dual-atom sites on hollow carbon enabling high-performance rechargeable zinc–air batteries
Source: Chem Sci. 2025 Nov 25;17(4):2155–63. doi: 10.1039/d5sc07448g (PMC12670757; doi:10.1039/d5sc07448g)
Supplement: SC-017-D5SC07448G-s001 [file SC-017-D5SC07448G-s001.pdf]

Supplementary Information (SI) for Chemical Science.
This journal is © The Royal Society of Chemistry 2025

Supporting Information

Synergistic Fe-Ni Dual-Atom Sites on Hollow Carbon Enabling High-Performance Rechargeable Zinc-Air Batteries

Yue Wang,[a, b] Jianhua Wang,[c] Xueting Feng,[a, b] Guanzhen Chen,[a, b] Xusheng Wang,[e] Tao Gan,*[d] Xing Fan,*[f] Haiping Lin[c] and Yunhu Han*[a, b]*

[a] State Key Laboratory of Flexible Electronics (LoFE), Institute of Advanced Materials (IAM), and School of Chemistry and Life Sciences, Nanjing University of Posts & Telecommunications, Nanjing, China. E-mail: iamyhhan@njupt.edu.cn; guanzhenchen123@163.com

[b] Frontiers Science Center for Flexible Electronics, and Institute of Flexible Electronics (IFE), Northwestern Polytechnical University, Xi'an, China.

[c] School of Physics and Information Technology, Shaanxi Normal University, Xi'an, China.

[d] Shanghai Synchrotron Radiation Facilities, Shanghai institute of Applied Physics, Chinese Academy of Sciences, Shanghai, China. E-mail: gant@sari.ac.cn

[e] State Key Laboratory of Bio-based Fiber Materials, School of Materials Science and Engineering, Zhejiang Sci-Tech University, Hangzhou, China.

[f] Center for Carbon-based Electronics and Key Laboratory for the Physics and Chemistry of Nanodevices, School of Electronics, Peking University, Beijing, China. E-mail: xingf@pku.edu.cn

† Y. Wang, J. H. Wang and X. T. Feng contributed equally to this work.

Experimental Procedures

Chemicals and reagents. Zinc nitrate hexahydrate ($Zn(NO_3)_2·6H_2O$, 99%), nickel nitrate hexahydrate ($Ni(NO_3)_2·6H_2O$, 99%), methanol, iron acetylacetone ($Fe(acac)_3$, 98%), 2-MeIM (2-MeIM, 98%), 1,4-benzenedicarboxylic acid (98%), zirconium chloride ($ZrCl_4$, 98%), 2-aminoterephthalic acid (98%), copper nitrate hexahydrate ($Cu(NO_3)_2·6H_2O$, 99%), isophthalic acid (98%), trimellitic acid (98%), N,N-dimethylformamide (DMF) and RuO_2 were purchased from Adamas. Zinc acetate dihydrate ($Zn(CH_3COO)_2$, 99%), anhydrous ethanol, concentrated hydrochloric acid (36-38%) and potassium hydroxide (KOH) were purchased from Greagent. Sodium formate (99%) purchased from Alfa Aesar. All reagents were used as received and did not need to be further purified. 20% Pt/C was purchased from Johnson Matthy, UK.

Preparation of Fe ZIF-8. 5.6 mmol of $Zn(NO_3)_2·6H_2O$, 1.2 mmol of $Fe(acac)_3$ and 22.5 mmol of 2-MeIM were dissolved in 45 mL of methanol, respectively, and stirred for 10 min. after waiting for complete dissolution, the two solutions were mixed. At room temperature, it was stirred at a stirring rate of 450 rpm for 20 h. The precipitated product was collected by washing and centrifugation with methanol solution. The product was put into a vacuum drying oven and kept under vacuum at 65 °C for 8 h. Finally, a yellow Fe ZIF-8 powder was obtained.

Synthesis of Fe ZIF-8@ZIF-8-NH$_2$. Appropriate amount of Fe ZIF-8 and $Zn(NO_3)_2·6H_2O$ were dissolved in 8 mL of DMF solution respectively. Afterwards, the two solutions were mixed well. 10 mL of aqueous solution dissolved with 2-MeIM and sodium formate was added slowly and the reaction was stirred for 1 h. The precipitate was collected by centrifugation with methanol.

Synthesis of FeNi-*h*CN. The obtained yellowish powder Fe ZIF-8@ZIF-8-NH$_2$ was raised to 920 °C under argon atmosphere at a heating rate of 5 °C per minute and kept at high temperature pyrolysis for 2 h to obtain a black powder. The Ni source was then introduced by impregnation, and the black powder was pyrolyzed at a low temperature of 500 °C under argon atmosphere for 2 h. The black powder was stirred in dilute hydrochloric acid overnight, and the diatomic electrocatalyst FeNi-*h*CN was obtained after washing and centrifugal drying.

Synthesis of Fe-*h*CN, Ni-*h*CN and FeNi-CN. Fe-*h*CN was obtained by pyrolysis of

Fe ZIF-8@ZIF-8-NH_2 at 920 °C and low-temperature calcination at 500 °C, followed by acid washing with dilute hydrochloric acid solution. Ni-*h*CN was obtained by coating ZIF-8 with ZIF-8-NH_2, and after pyrolysis at 920 °C, Ni source was added and the second calcination was carried out. Similarly, Ni-*h*CN needs acid cleaning. While FeNi-CN is directly utilized as a black powder from the high temperature pyrolysis of Fe ZIF-8 without the synthesis of the second layer of ZIF-8-NH_2. After impregnation with Ni source, the process of secondary calcination and acid washing is consistent with other samples.

Preparation of UiO-66 system. Add 0.205 g 1,4-benzenedicarboxylic acid to 10 ml N,N-dimethylformamide (DMF) solution, stir for 25 min, dissolve before adding 2.5 mL of acetic acid solution. Add 0.318 g of $ZrCl_4$ to 10 ml DMF solution, sonicate to dissolve and mix with the previous solution, stir for 25 min. hydrothermal at 120 °C for 24 h. centrifuge with ethanol and dry. UiO-66-NH_2 package of UiO-66: 100 mg of UiO-66 powder was taken and added to 50 ml of DMF solution containing PVP, after which 30 ml of DMF solution containing 2-aminoterephthalic acid (0.1225 g) and 20 ml of DMF solution containing $ZrCl_4$ (0.157 g) and 20 ml of acetic acid were added sequentially. The mixture was stirred well and heated with stirring at 120 °C for 12 h, followed by centrifugation with a mixture of methanol and DMF solution. The solid obtained was burned for 2 h at 900 °C under argon atmosphere.

Preparation of HKUST-1 system. 1.82 g of copper nitrate was dissolved in 50 mL of methanol. 0.437 g of trimellitic acid was dissolved in 50 mL of methanol, and the two solutions were mixed and stirred at room temperature for 2 hours, then centrifuged to collect the precipitate. Defect HKUST-1 package HKUST-1: Disperse the precipitate obtained above in 30 mL of methanol, then sequentially add a mixture of isophthalic acid and trimellitic acid (0.875 g, molar ratio 4:2) in methanol solution and 30 mL of methanol solution containing 0.91 g of copper nitrate. Centrifuge and collect the precipitate. The solid obtained was burned for 2 h at 900 °C under argon atmosphere.

Structural characterizations. Scanning electron microscopy (SEM) was performed on a Hitachi S4800 electron microscope with an accelerating voltage of 30 kV. Transmission electron microscopy (TEM) was performed on a Hitachi H-800

microscope. The prepared catalysts were characterized by high resolution transmission electron microscopy (HR-TEM) as well as high angle annular dark field energy dispersive spectroscopy (HAADF-EDS, JEOL-2100FFETEM, operating voltage 200 kV). Powder X-ray diffractometry (PXRD) experiments were performed using a Rigaku RU-200b X-ray powder diffractometer with Cu Kα rays (λ = 1.5406 Å), 2° min^{-1}. Raman spectra were collected on a Horiba JOBIN YVON US/HR800 UV-type high-performance micro-Raman spectrometer system with a laser wavelength of 632.8 nm. X-ray photoelectron spectroscopy (XPS) analysis was performed using a ULVAC PHI Quantera microscope analyzer.

Electrochemical measurements. Scan rate of 50 mV/s with a suitable potential range, and LSV plots were recorded at a scan rate of 10 mV/s with a suitable potential range. Tafel plots were obtained from the transformation of LSV test curves, and the kinetic current densities and Koutecky-Levich (K-L) plots were obtained on the basis of the following equations:

$$\frac{1}{J} = \frac{1}{J_k} + \frac{1}{J_L} = \frac{1}{Bw^{1/2}} + \frac{1}{J_k}$$

$$B = 0.62nFC_0D_0^{2/3}V^{-1/6}$$

where J is the test current, J_k and J_L are the kinetic and limiting currents, w is the angular velocity of the disk, n is the number of electron transfers, F is the Faraday's constant (96485 C mol^{-1}), C_0 is the volumetric concentration of O_2 (1.2×10^{-6} mol cm^{-3}), and D_0 is the kinematic viscosity of the electrolyte (0.01 cm^2 s^{-1}).

Accelerated Durability Testing (ADT) for oxygen reduction reactions was conducted in a saturated oxygen 0.1 M KOH electrolyte solution. Cyclic potential scans were performed at a scan rate of 50 mV s^{-1} between 0.3 and -0.6 V.

Then, the transferred electron number (n) was calculated with the following equation:

$$n = 4 \times I_d/(I_d + I_r/N_c)$$

and H_2O_2 yield was calculated with the formular of

$$H_2O_2\% = 200\ I_r/N_c/(I_d + I_r/N_c)\%$$

where I_d, I_r and N_c are the disk current, ring current and the collection efficiency of the

ring disk electrode (0.424), respectively.

Density function theory calculations: All the density functional theory (DFT) calculations were conducted based on the Vienna *ab-inito* Simulation Package (VASP).[1-3] The frozen-core projector augmented-wave (PAW) potentials was employed for the core and valence electronic interactions.[4] Our exchange-related functional adopts the Perdew Burke-Ernzerhof (PBE) under the generalized gradient approximation (GGA) method.[5-7] The FeNi-*h*CN, Fe-*h*CN and Ni-*h*CN were constructed as models for theoretical calculations. The energy cut-off was set to 400 eV. The DFT-D3 method by Gimme was employed to calculate the van der Waals interaction.[8] The Brillouin zone was sampled by a 2 × 2 × 1 Γ-centered k-point grid. A vacuum region in the *z*-direction was set as 30 Å to avoid interactions between the periodic images. The convergence threshold was set as 1×10^{-4} eV in energy and 0.01 eV per Angstrom in force.

The free energy of each adsorbed intermediates (G) can be written as:

$$G = E + E_{ZPE} - TS$$

Where E is the total energy, obtained from the DFT calculation. E_{ZPE} is the zero-point energy, T is 298.15 K in this study and S is the entropy. The adsorption free energy of different catalytic intermediates can be calculated based on the computational hydrogen electrode (CHE) model proposed by Nørskov and his co-workers.[20]

$$\Delta G_{*OH} = G_{*OH} + 1/2G_{H_2} - G_{*} - G_{H_2O}$$

$$\Delta G_{*O\text{-}*H_2O} = G_{*O\text{-}*H_2O} + G_{H_2} - G_{*} - 2G_{H_2O}$$

$$\Delta G_{*OOH} = G_{*OOH} + 3/2G_{H_2} - G_{*} - 2G_{H_2O}$$

$$\Delta G_{*OH-*OH} = G_{*OH-*OH} + G_{H_2} - G_{*} - 2G_{H_2O}$$

$$\Delta G_{*O-*OH} = G_{*O-*OH} + 3/2G_{H_2} - G_{*} - 2G_{H_2O}$$

$$\Delta G_{*O-*O} = G_{*O-*O} + 2G_{H_2} - G_{*} - 2G_{H_2O}$$

$$\Delta G_{*O_2} = G_{*O_2} + 2G_{H_2} - G_{*} - 2G_{H_2O}$$

$$\Delta G_{*H_2O_2} = G_{*H_2O_2} + G_{H_2} - G_{*} - 2G_{H_2O}$$

Where G_{*}, G_{*OH}, $G_{*O\text{-}*H_2O}$, G_{*OOH}, $G_{*OH-*OH}$, G_{*O-*OH}, G_{*O-*O}, G_{*O_2} and $G_{*H_2O_2}$ are the free energies of the catalyst, the *OH, *O-*H_2O, *OOH, *OH-OH, *O-*OH, *O-*O, *O_2 and *H_2O_2 intermediates, respectively. G_{H_2O} and G_{H_2} are energies of isolated H_2O and H_2 molecules. The free energy of the O_2 molecule is derived as $G_{O_2} = 2G_{H_2O} - 2G_{H_2} + 4.92\ \text{eV}$, because the O_2 molecule in the high-spin ground state is poorly described in DFT calculations.[21] Consequently, the Gibbs free energy change (ΔG) for each reaction step in the AEM-OER is calculated using the following equation is calculated as:

$$\Delta G_1 = \Delta G_{*OH}$$

$$\Delta G_2 = \Delta G_{*O\text{-}*H_2O} - \Delta G_{*OH}$$

$$\Delta G_3 = \Delta G_{*OOH} - \Delta G_{*O\text{-}*H_2O}$$

$$\Delta G_4 = \Delta G_{*O_2} - \Delta G_{*OOH}$$

$$\Delta G_5 = 4.92\ \text{eV} - \Delta G_{*O_2}$$

And for OPM-OER, the Gibbs free energy of the elementary steps (ΔG) is given by following expression:

$$\Delta G_1 = \Delta G_{*OH}$$

$$\Delta G_2 = \Delta G_{*OH\text{-}*OH} - \Delta G_{*OH}$$

$$\Delta G_3 = \Delta G_{*O\text{-}*OH} - \Delta G_{*OH\text{-}*OH}$$

$$\Delta G_4 = \Delta G_{*O-*O} - \Delta G_{*O\text{-}*OH}$$

$$\Delta G_5 = \Delta G_{*O_2} - \Delta G_{*O-*O}$$

$$\Delta G_6 = 4.92\ \text{eV} - \Delta G_{*O_2}$$

While the four-electron ORR pathway is the microscopic reverse of the AEM-OER process in terms of free energy changes, the corresponding steps for the two-electron pathway are provided as follows:

$$\Delta G_1 = 4.92\ \text{eV} - \Delta G_{*O_2}$$

$$\Delta G_2 = \Delta G_{*O_2} - \Delta G_{*OOH}$$

$$\Delta G_3 = \Delta G_{*H_2O_2} - \Delta G_{*OOH}$$

Quasi-solid-state Zn-air battery assembly. In a conventional setup for the solid-state Zn-air battery, a polished zinc foil measuring 2 × 2 cm^2 (with an additional 2 × 2 cm^2 left blank for current collection) was utilized as the anode. Catalysts coated on carbon cloth measuring 2 × 2 cm^2 (with an additional 2 × 2 cm^2 left blank for current collection) were pressed onto the top of a gel polymer to serve as the cathode. Subsequently, two pieces of white breathable tape were employed to seal the device. Regarding the preparation of the catalyst inks, a mixture was created by blending 10 mg of the FeNi-*h*CN catalyst with 985 μL of ethanol and 15 μL of Nafion solution (5 wt.%) through sonication for 30 minutes. In contrast, for the commercial precious metal catalysts (Pt/C: RuO_2 = 1:1, molar ratio), 5 mg of the catalyst and 15 μL of 5 wt.% Nafion solution were dispersed in 985 μL of ethanol using sonication for 30 minutes. The mass loading of the catalysts was determined to be 2.50 mg cm^{-2} for the FeNi-*h*CN-based battery and 1.25 mg cm^{-2} for the Pt/C+RuO_2-based battery. The gel polymer electrolyte was prepared as follows: 4 g of polyvinyl alcohol powder was dissolved in 40 mL of deionized water at 95 °C with stirring until a homogeneous and transparent solution was achieved. Subsequently, 4 mL of 18 M KOH was added dropwise over 40 minutes at 95°C with continuous stirring. The solution was then poured into a mold and frozen in a freezer at -20 °C for 2 hours followed by 0 °C for 4 hours. The long-term cycling test was performed without replacement of the electrolyte or Zn anode throughout the entire process.

Liquid Zn-air battery assembly. A homemade, uncomplicated alkaline/neutral liquid Zn-air battery configuration was assembled, employing carbon paper coated with the FeNi-*h*CN catalyst as the air cathode, Zn foil as the anode (the thickness is 0.05 mm),

6 M KOH and 0.2 M $Zn(CH_3COO)_2$ as the alkaline electrolyte. The interface area between the catalyst and the electrolyte on the carbon paper was measured at 1 cm^2, with a mass loading of the FeNi-*h*CN catalyst on the carbon paper estimated at approximately 2 mg cm^{-2}. Additionally, a control sample was prepared by mixing Pt/C and RuO_2 at a molar ratio of 1:1. The specific capacity of the battery can be calculated using the equation:

Specific Capacity = [Discharge Current (mA) × Time (h)]/ Weight of consumed Zn (g)

Energy Density = {[Current (mA) × Time (h)] /Weight of consumed Zn (kg)} ×Discharge

Supplementary Figures and Tables

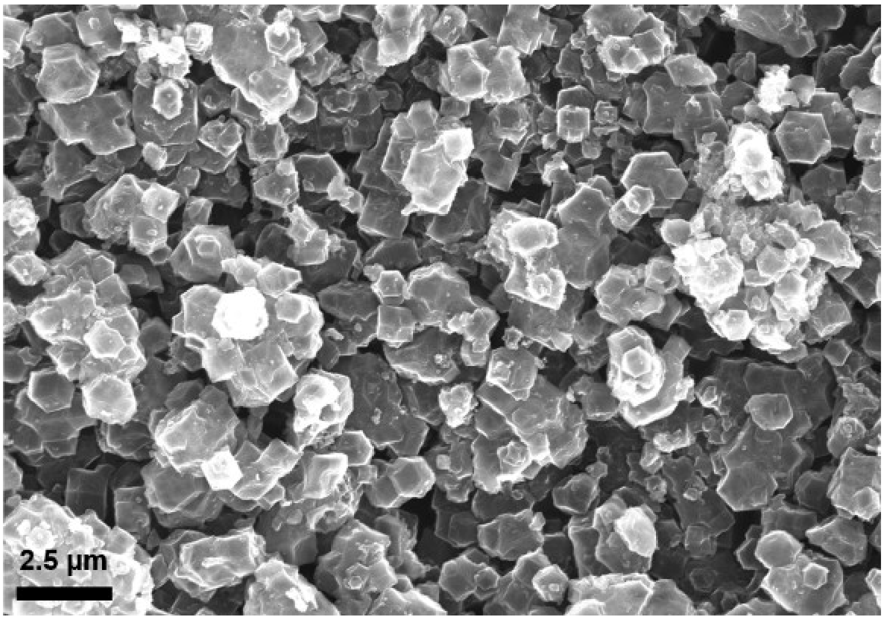

Figure S1. SEM images of the prepared FeNi-*h*CN catalyst.

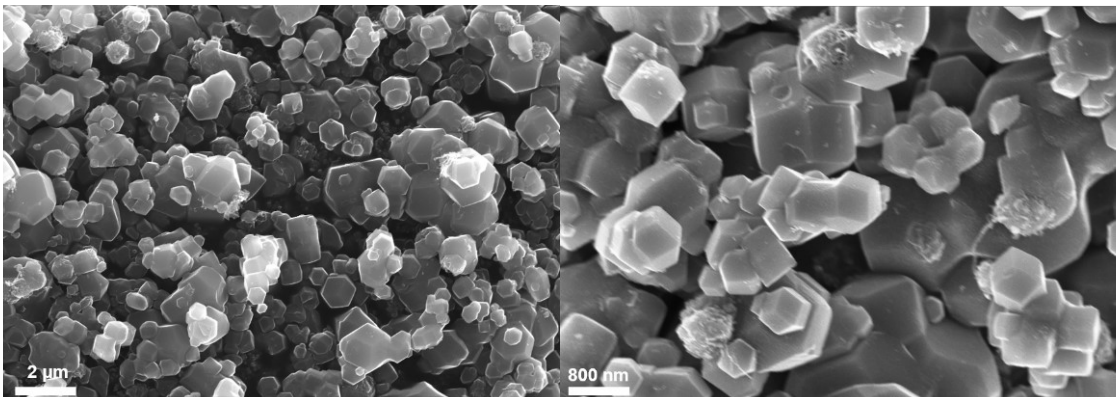

Figure S2. SEM images of the prepared FeNi-CN catalyst.

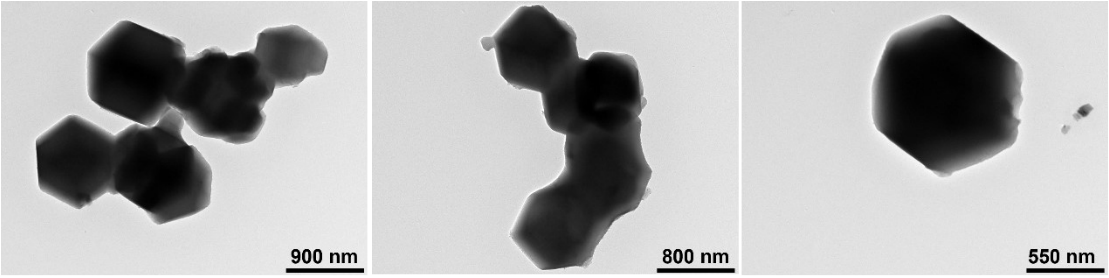

Figure S3. TEM images of the prepared FeNi-CN catalyst.

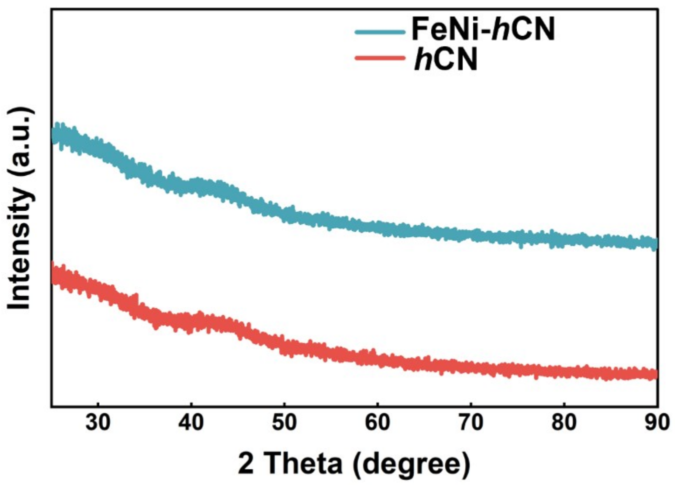

Figure S4. PXRD patterns of the FeNi-*h*CN and *h*CN catalysts.

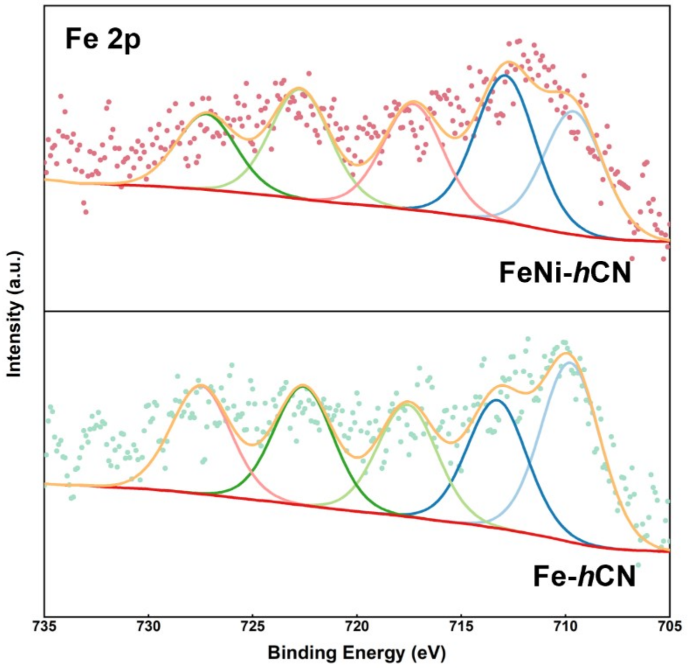

Figure S5. Fe 2p spectra of the FeNi-*h*CN and Fe-*h*CN catalysts.

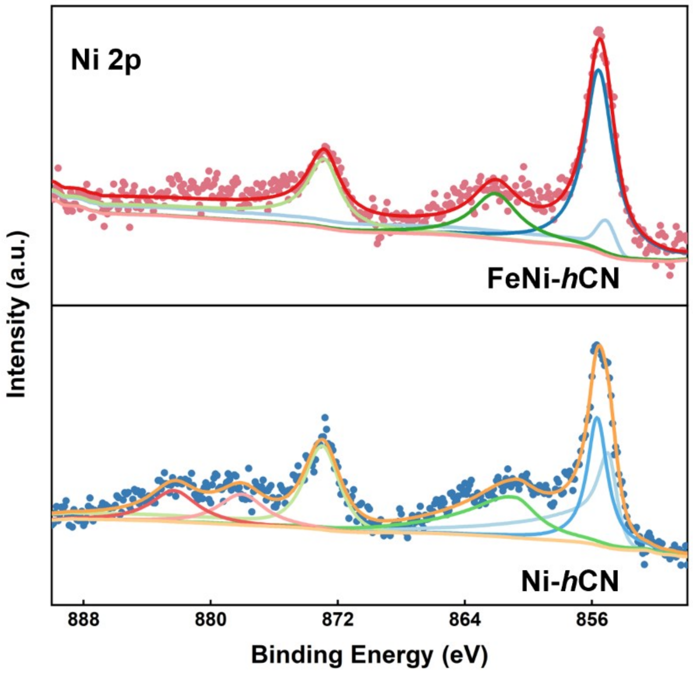

Figure S6. Ni 2p spectra of the FeNi-*h*CN and Ni-*h*CN catalysts.

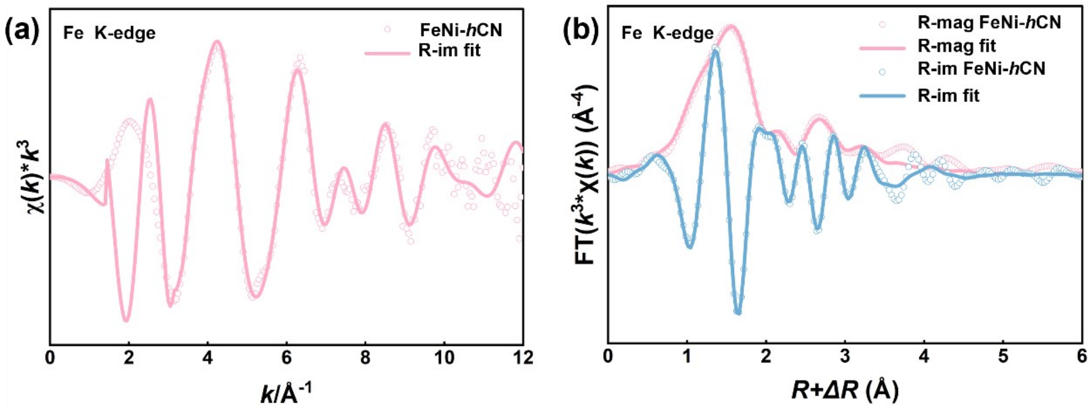

Figure S7. EXAFS fitting curves of Fe K-edge at *k* space for the FeNi-*h*CN catalyst.

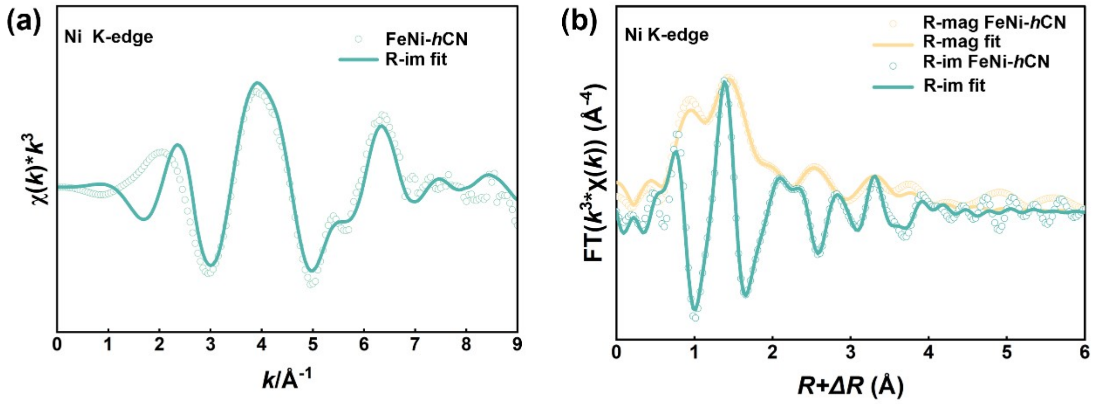

Figure S8. EXAFS fitting curves of Ni K-edge at *k* space for the FeNi-*h*CN catalyst.

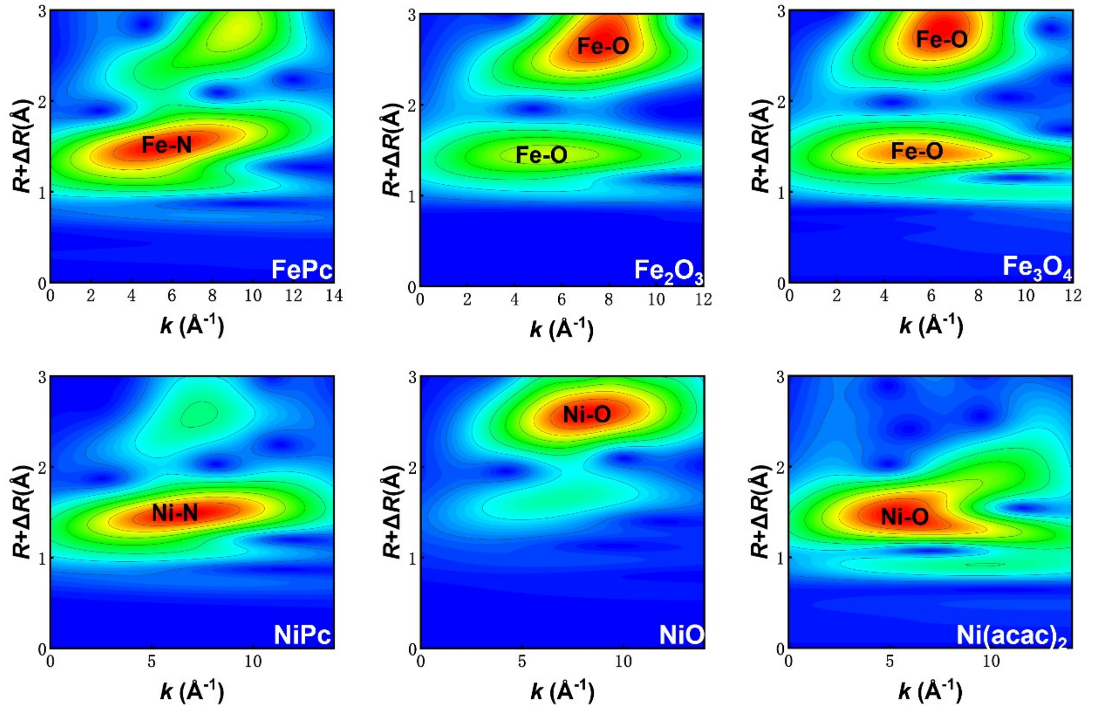

Figure S9. WT EXAFS contour plots of Fe K-edge for FePc, Fe_2O_3 and Fe_3O_4, Ni K-edge for NiPc, NiO and $Ni(acac)_2$.

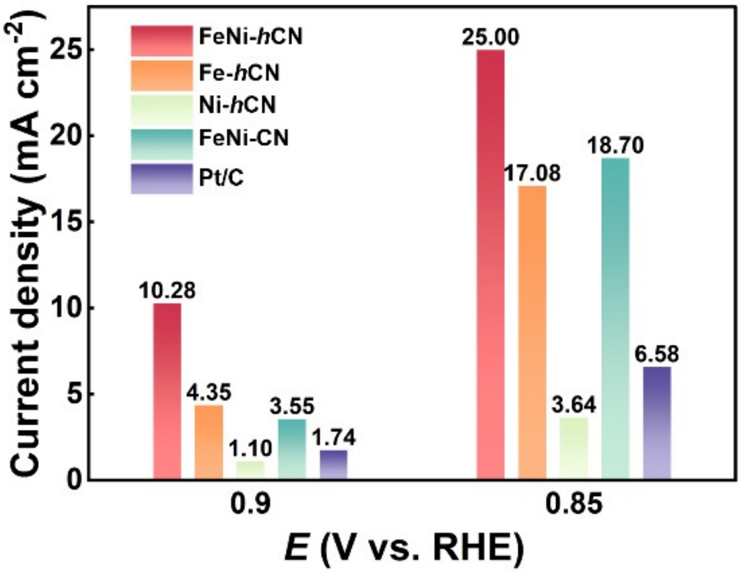

Figure S10. J_k of samples at 0.9 V and 0.8 V voltage for FeNi-*h*CN and the controls.

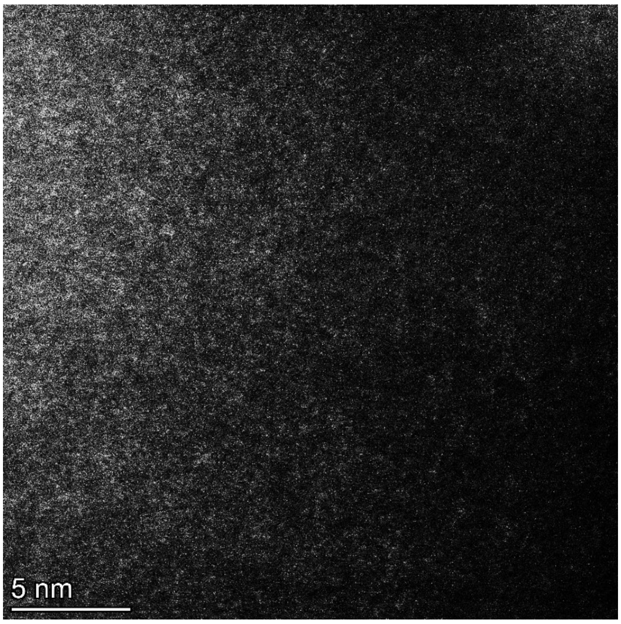

Figure S11. HAADF-STEM image of FeNi-*h*CN after stabilization.

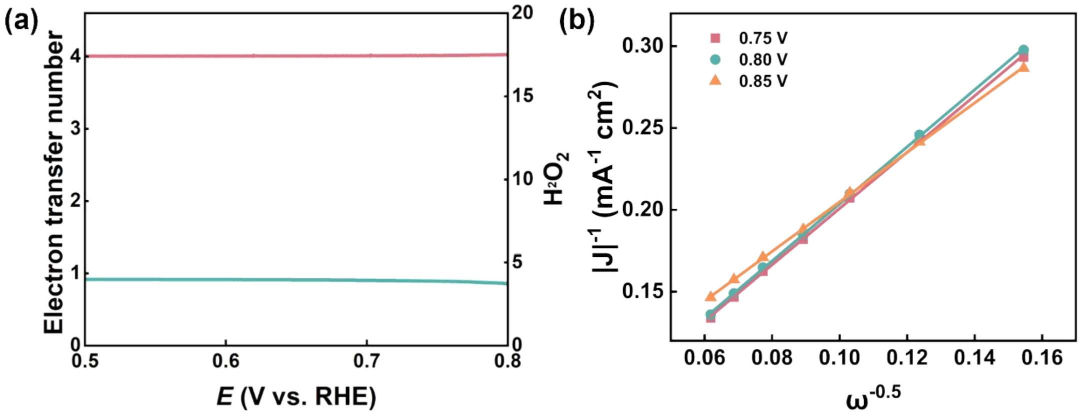

Figure S12. (a) Electron transfer number and peroxide yield derived from ring-disk voltammograms of FeNi-*h*CN in O_2 saturated 0.1 M KOH media and (b) Koutecky-Levich (K-L) plots of the FeNi-*h*CN catalyst in O_2-saturated 0.1 M KOH media.

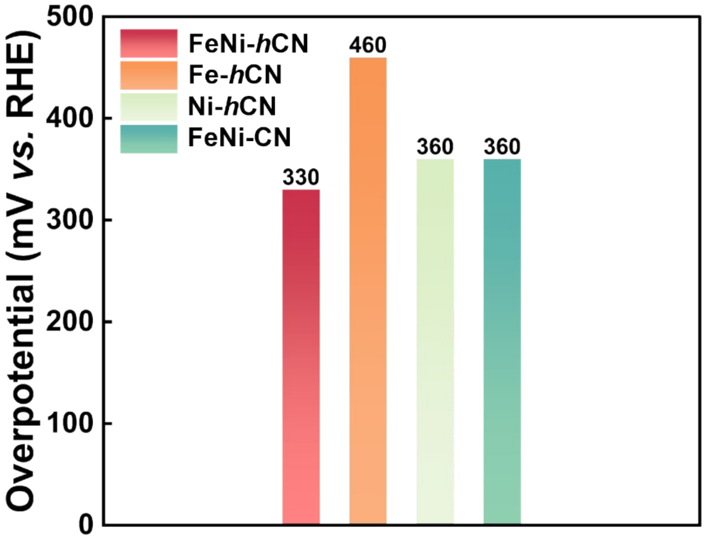

Figure S13. Comparison of overpotential@10 mA cm^{-2}.

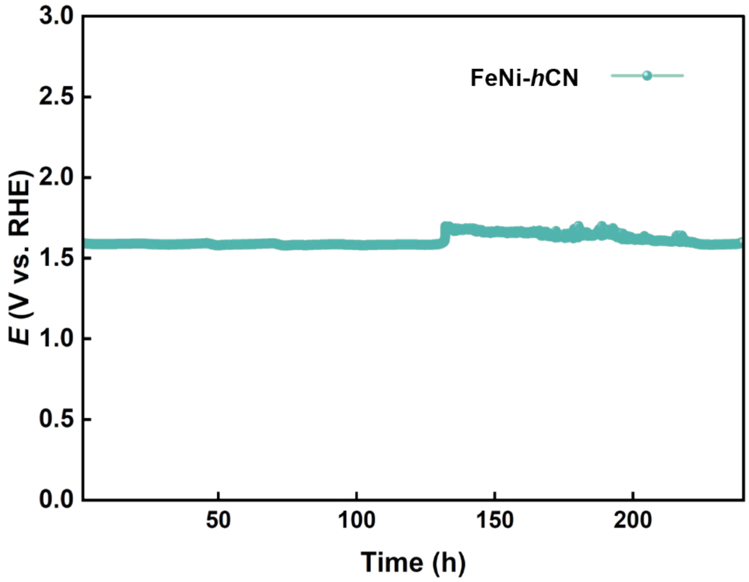

Figure S14. Chronopotentiometry test for the FeNi-*h*CN in N_2 saturated 1 M KOH media.

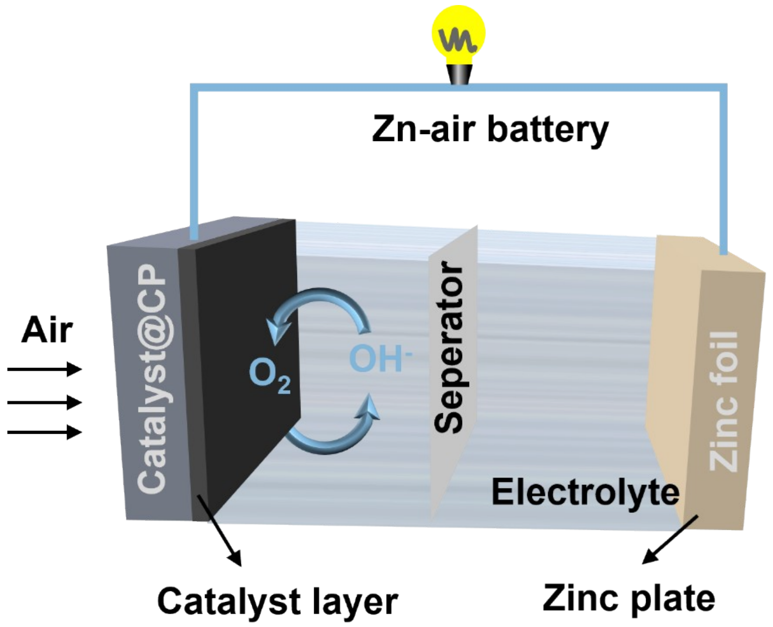

Figure S15. Schematic illustration of a homemade liquid alkaline ZAB.

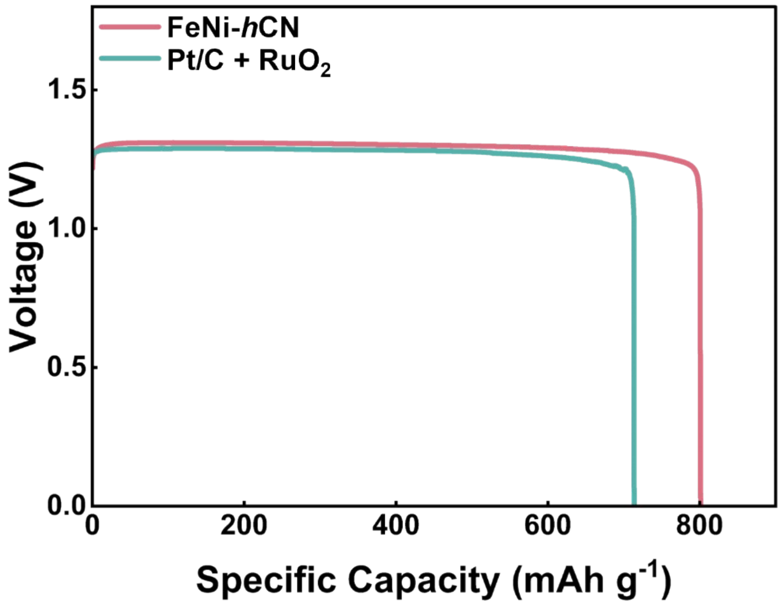

Figure S16. Constant current discharge curve of liquid ZAB at 5 mA·cm^{-2}.

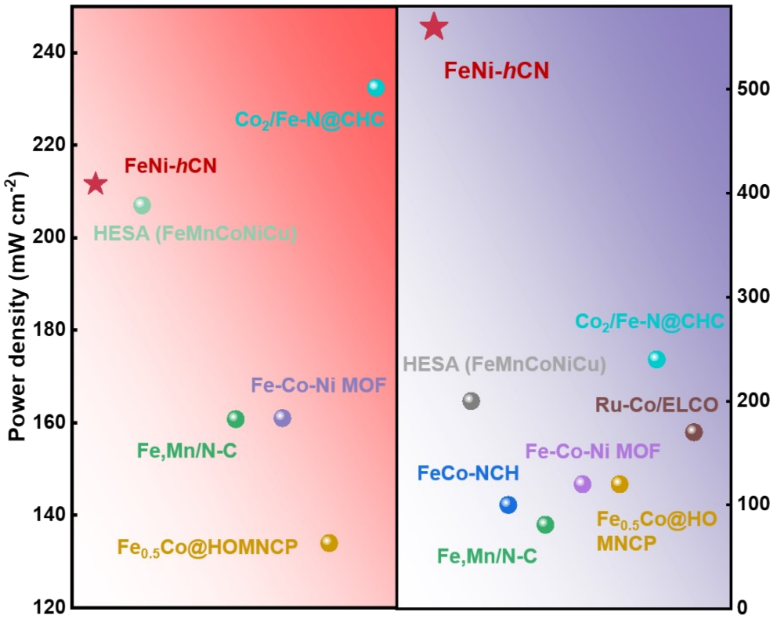

Figure S17. Performance comparison of FeNi-*h*CN-based ZAB and the reported non-precious metal-based liquid ZABs.

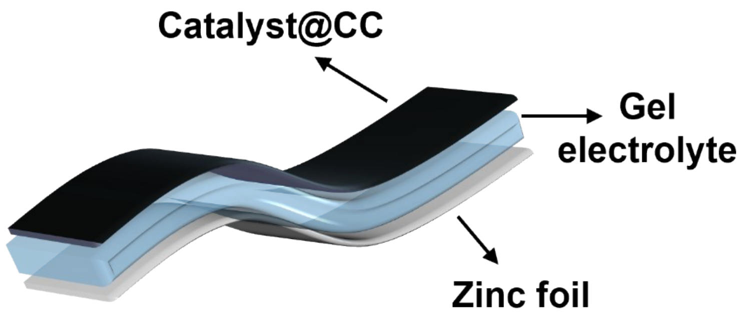

Figure S18. Schematic illustration of a homemade flexible quasi-solid-state alkaline ZAB.

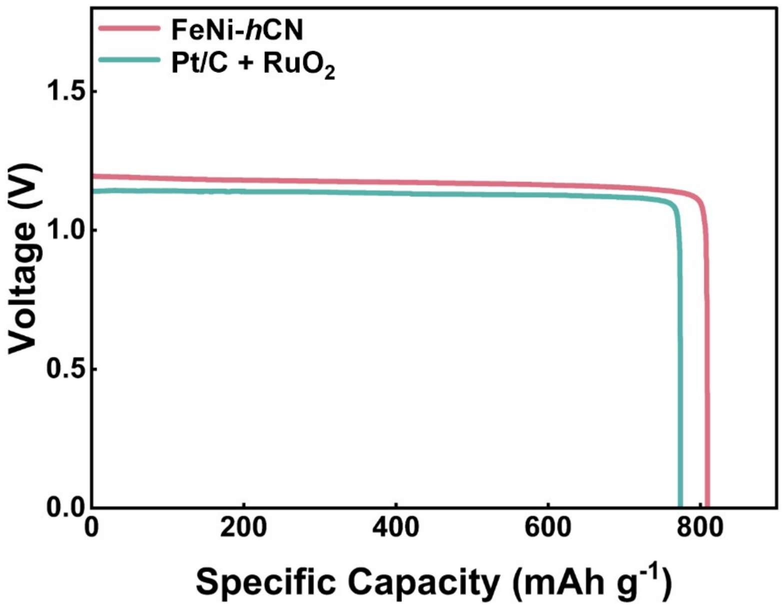

Figure S19. Constant current discharge curve of flexible quasi-solid-state ZAB at 5 mA·cm^{-2}.

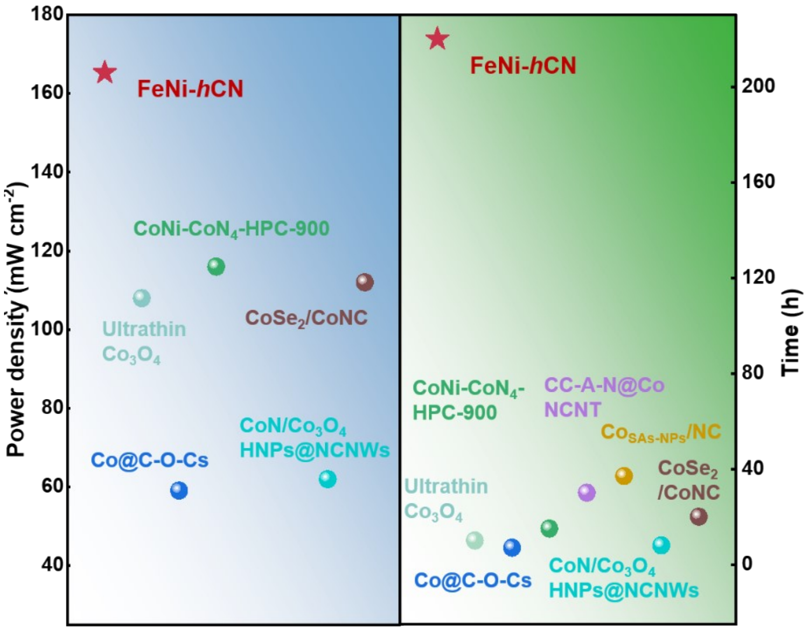

Figure S20. Performance comparison of FeNi-*h*CN-based flexible quasi-solid-state ZABs and the reported non-precious metal-based flexible quasi-solid-state ZABs.

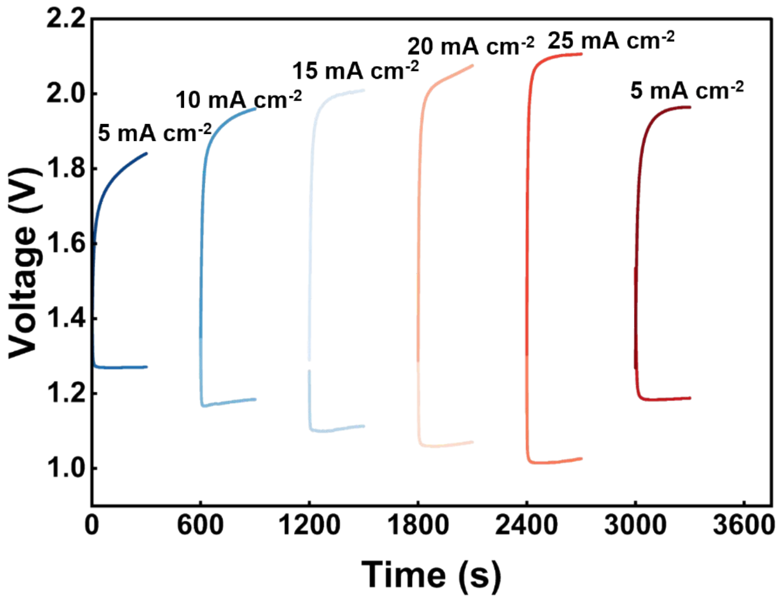

Figure S21. Charge/discharge voltage gap of flexible quasi-solid-state ZAB with different current densities.

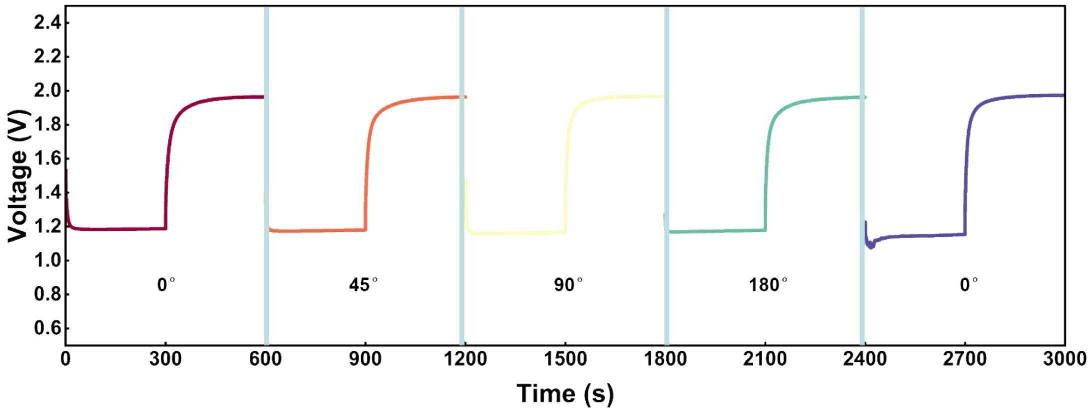

Figure S22. Cycling stability of flexible quasi-solid-state ZAB at different bending angles.

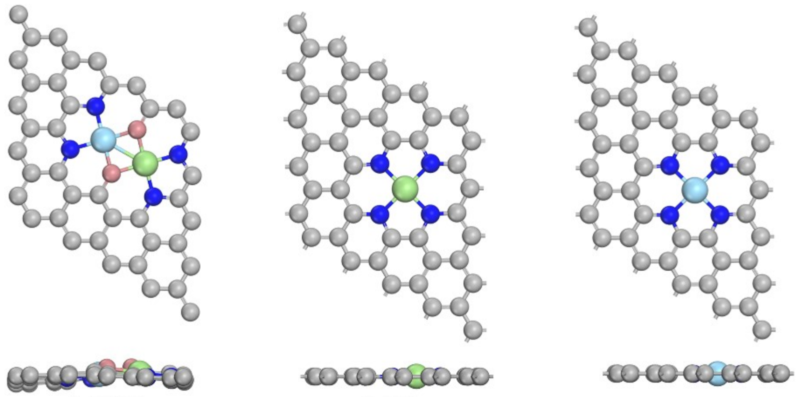

Figure S23. Top and side views of optimized configurations of FeNi-*h*CN, Fe-*h*CN, and Ni-*h*CN.

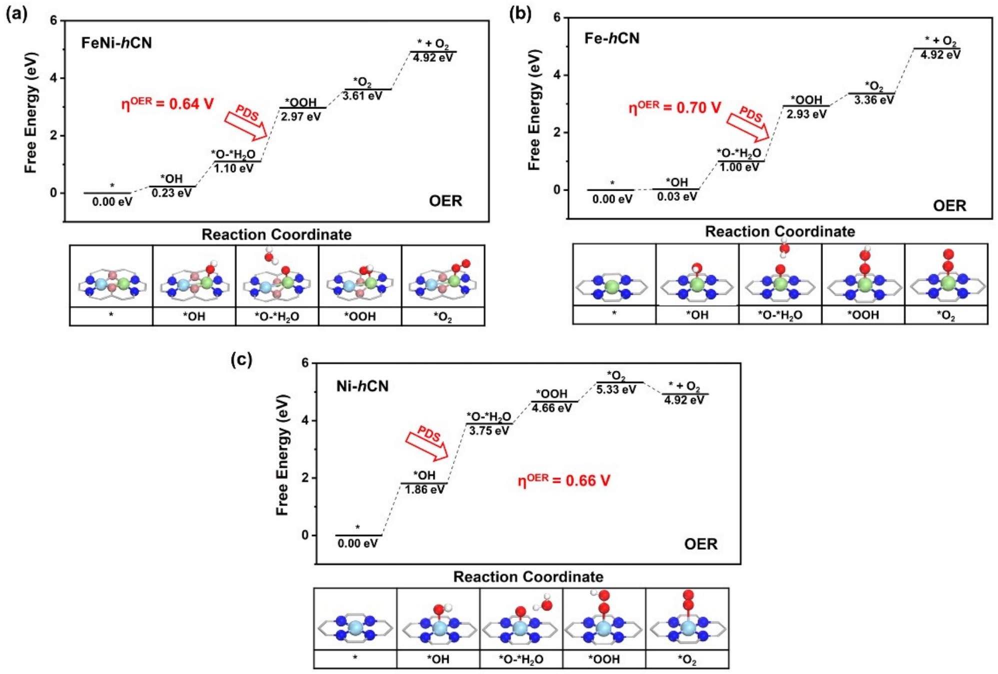

Figure S24. Gibbs Free energy diagrams of AEM-OER pathways on (a) FeNi-*h*CN, (b) Fe-*h*CN and (c) Ni-*h*CN.

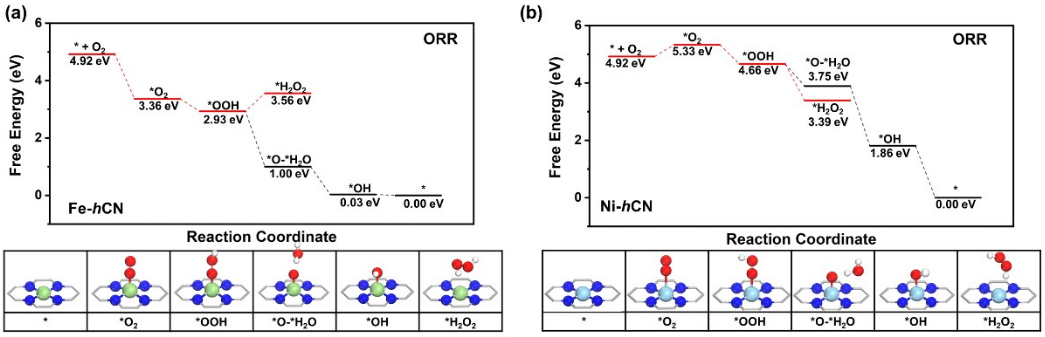

Figure S25. Gibbs Free energy diagrams of ORR pathways on (a) Fe-*h*CN and (b) Ni-*h*CN.

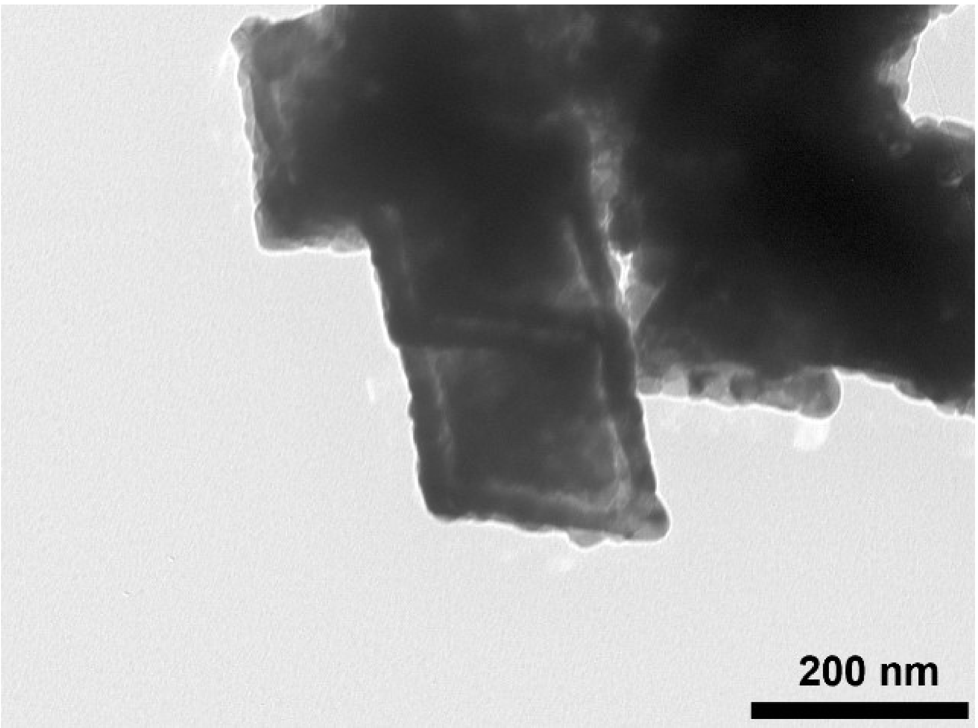

Figure S26. TEM image of the prepared UiO-66 system.

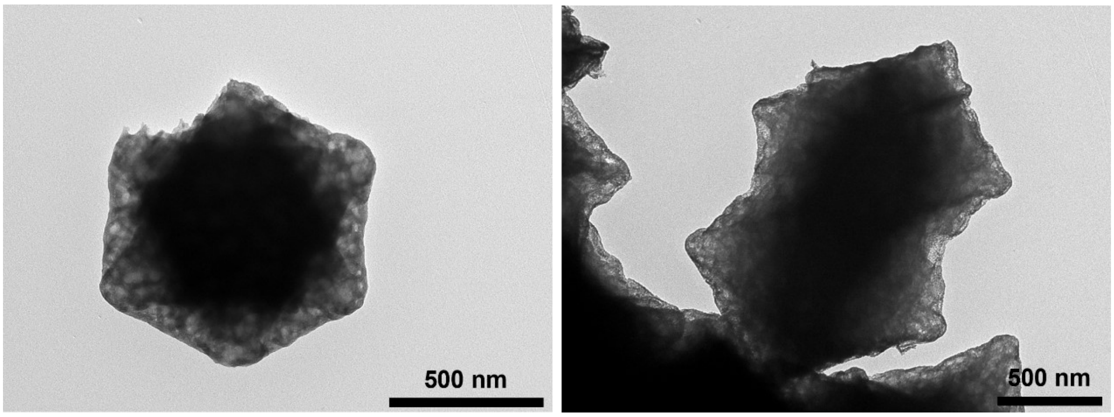

Figure S27. TEM images of the prepared HKUST-1 system.

Table S1. Content of metal elements in the FeNi-*h*CN catalyst.

Sample	Element	Content (wt%)
FeNi-*h*CN	Fe<br>Ni	1.7<br>1.3

Table S2. Results of the analysis of the relative XPS content of N species in the samples.

Sample	Pyridinic N	M-N	Pyrrolic N	Graphitic N	Oxidized N
FeNi-*h*CN	43.92%	19.05%	24.24%	5.19%	8.23%
Fe-*h*CN	35.84%	12.90%	29.75%	18.18%	3.23%
Ni-*h*CN	37.88%	14.77%	27.27%	8.33%	11.74%

Table S3. Structural parameters of FeNi-*h*CN extracted from the EXAFS fitting. (S_0^2=1.0).

Sample	Shell	CN	R (Å)	σ^2 (10^{-3} Å^2)	ΔE_0 (eV)	R-factor
FeNi-*h*CN	Fe-N	2.904±0.591	1.824±0.126	0.231±0.119	7.934	0.0096
	Fe-Ni	1.26±1.737	2.553±0.0935	0.146±0.0256		
	Ni-N	3.71±0.91	1.991±0.027	1.3±0.14	-5.755	0.016
	Ni-Fe	0.15±0.04	2.545±0.04	3.1±0.3		

S_0^2 is the amplitude reduction factor,

CN is the coordination number,

R is interatomic distance (the bond length between central atoms and surrounding coordination atoms),

σ^2 is Debye-Waller factor (a measure of thermal and static disorder in absorber-scatterer distances),

ΔE_0 is edge-energy shift (the difference between the zero kinetic energy value of the sample and that of the theoretical model),

R-factor is used to value the goodness of the fitting.

Table S4. Summary of the performance of the non-noble metal bifunctional catalysts

Samples	$E_{1/2}$ (V *vs.* RHE)	E_{j10} (V *vs.* RHE)	Δ*E*	Refs.
**FeNi-*h*CN**	**0.91**	**1.56**	**0.65**	**This work**
$NiCo_{1.8}Fe_{0.2}O_4$@NCF	0.86	1.50	0.64	[9]
PtFeCoNiMoY/CNT	0.758	1.468	0.71	[10]
Co, Fe-DACs/NCs	0.871	1.588	0.717	[11]
FeCoNiMoW	0.703	1.463	0.76	[12]
Co-N_5	0.92	1.50	0.58	[13]
S-CFZ	0.85	1.53	0.68	[14]
CoO_x@CS	0.80	1.60	0.80	[15]
FeCo-MI@TAP-900	0.86	1.615	0.755	[16]
FeCo–N_3O_3@C	0.936	1.56	0.624	[17]
FeNC@LDH	0.90	1.47	0.57	[18]
COP_{BTC}-M	0.945	1.735	0.79	[19]

References

1. G. Kresse and J. Hafner, *Phys. Rev. B*, 1993, **48,** 13115-13118.
2. G. Kresse, J. Furthmüller, *Comput. Mater. Sci.* 1996, **6,** 15-50.
3. G. Kresse, J. Furthmüller, *Phys. Rev. B*, 1996, **54,** 11169-11186.
4. P. E. Blöchl, Physical review. B, 1994, **50,** 17953-17979.
5. J. P. Perdew, Y. Wang, *Phys. Rev. B*, *r* 1992, **45,** 13244-13249.
6. J. P. Perdew, J. A. Chevary, D. J. Vosko, K. A. Jackson, M. R. Pederson, D. J. Singh and C. Fiolhais, *Phys. Rev. B*, 1992, **46,** 6671-6687.
7. J. P. Perdew, K. Burke, Y. Wang, *Phys. Rev. B*, 1996, **54,** 16533-16539.
8. S. Grimme, S. Ehrlich, L. Goerigk, *J. Comput. Chem.* 2011, **32,** 1456-1465.
9. Y. Liu, L. Zhou, S. Liu, S. Li, J. Zhou, X. Li, X. Chen, K. Sun, B. Li, J. Jiang, H. Pang, *Angew. Chem. Int. Ed.* 2024, **63,** e202319983.
10. J. Han, W. Zhang, K. Liu, H. Zheng, Y. Li, L. Luo, S. Gong, Y. Jia, X. Liang, *Appl. Surf. Sci.* 2025, **687,** 162238.
11. Z. Lu, Z. Wang, Z. Yang, X. Jin, L. Tong, R. J. Xu, K. Kong, Y. Zhang, Y. Wang, Y. Liu, L. Meng, Z. Pan, S. J. Hwang, L. Li, *Adv. Funct. Mater.* 2025, **35,** 2418489.
12. R. He, L. Yang, Y. Zhang, D. Jiang, S. Lee, S. Horta, Z. Liang, X. Lu, A. Ostovari Moghaddam, J. Li, M. Ibáñez, Y. Xu, Y. Zhou, A. Cabot, *Adv. Mater.* 2023, **35,** 2303719.
13. Q. Luo, K. Wang, Q. Zhang, W. Ding, R. Wang, L. Li, S. Peng, D. Ji, X. Qin, *Angew. Chem. Int. Ed.* 2025, **64,** e202413369.
14. Y. Jiang, Y. P. Deng, R. Liang, N. Chen, G. King, A. Yu, Z. Chen, *J. Am. Chem. Soc.* 2022, **144,** 4783–4791.
15. M. Yang, S. Ding, X. Shu, W. Pan, J. Zhang, *Mater. Chem. Front.* 2022, **6,** 3706–3715.
16. J. Zheng, G. Hu, B. Liu, Y. Liu, H. Li, H. Zhao, M. Yang, *Energy Storage Mater.* 2024, **65,** 103106.
17. B. Tang, Y. Zhou, Q. Ji, Z. Zhuang, L. Zhang, C. Wang, H. Hu, H. Wang, B. Mei, F. Song, S. Yang, B. M. Weckhuysen, H. Tan, D. Wang, W. Yan, *Nat. Synth.* 2024, **3,** 878–890.

18. J. N. Liu, C. X. Zhao, J. Wang, X. Q. Fang, C. X. Bi, B. Q. Li, Q. Zhang, *Joule* 2024, **8,** 1804–1819.
19. C. Mi, H. Yu, L. Han, L. Zhang, L. Zhai, X. Li, Y. Liu, Z. Xiang, *Adv. Funct. Mater.* 2023, **33,** 2303235.
20. J. K. Norskov, J. Rossmeisl, A. Logadottir, L. Lindqvist, J. R. Kitchin, T. Bligaard and H. Jónsson, *Journal of Physical Chemistry B*, 2004, **108**, 17886-17892.
21. E. Sargeant, F. Illas, P. Rodríguez and F. Calle-Vallejo, *Journal of Electroanalytical Chemistry*, 2021, **896**, 115178.

Author Contributions

Y. Wang, J. Wang, and X. Feng contributed equally to this work. Y. Wang designed and performed the material synthesis, data analysis, and manuscript writing. J. Wang, X. Fang and H. Lin performed DFT calculations. X. Wang performed the HRTEM measurements. T. Gan performed the XAS measurement and data analysis. G. Chen assisted with electrochemical testing and data analysis. Y. Han proposed the idea, review and editing for this study.
